# Diversity of phenotypically non-dermatophyte, non-*Aspergillus* filamentous fungi causing nail infections: importance of accurate identification and antifungal susceptibility testing

**DOI:** 10.1080/22221751.2019.1598781

**Published:** 2019-04-02

**Authors:** Chi-Ching Tsang, James Y. M. Tang, Ka-Fai Chan, Chun-Yi Lee, Jasper F. W. Chan, Antonio H. Y. Ngan, Mei Cheung, Eunice C. L. Lau, Xin Li, Ricky H. Y. Ng, Christopher K. C. Lai, Kitty S. C. Fung, Susanna K. P. Lau, Patrick C. Y. Woo

**Affiliations:** a Department of Microbiology, Li Ka Shing Faculty of Medicine, The University of Hong Kong, Pokfulam, Hong Kong; b State Key Laboratory of Emerging Infectious Diseases, The University of Hong Kong, Pokfulam, Hong Kong; c Carol Yu Centre for Infection, The University of Hong Kong, Pokfulam, Hong Kong; d Collaborative Innovation Centre for Diagnosis and Treatment of Infectious Diseases, The University of Hong Kong, Hong Kong; e Department of Pathology, Queen Elizabeth Hospital, Pokfulam, King's Park, Hong Kong; f Department of Pathology, United Christian Hospital, Kwun Tong, Hong Kong

**Keywords:** Onychomycosis, non-dermatophyte, non-*Aspergillus*, molecular identification, antifungal susceptibility

## Abstract

Onychomycosis is most commonly caused by dermatophytes. In this study, we examined the spectrum of phenotypically non-dermatophyte and non-*Aspergillus* fungal isolates recovered over a 10-year period from nails of patients with onychomycosis in Hong Kong. A total of 24 non-duplicated isolates recovered from 24 patients were included. The median age of the patients was 51 years, and two-thirds of them were males. One-third and two-thirds had finger and toe nail infections respectively. Among these 24 nail isolates, 17 were confidently identified as 13 different known fungal species, using a polyphasic approach. These 13 species belonged to 11 genera and ≥9 families. For the remaining seven isolates, multilocus sequencing did not reveal their definite species identities. These seven potentially novel species belonged to four different known and three potentially novel genera of seven families. 33.3%, 41.7% and 95.8% of the 24 fungal isolates possessed minimum inhibitory concentrations of >1 µg/mL to terbinafine, itraconazole and fluconazole, respectively, the first line treatment of onychomycosis. A high diversity of moulds was associated with onychomycosis. A significant proportion of the isolates were potentially novel fungal species. To guide proper treatment, molecular identification and antifungal susceptibility testing should be performed for these uncommonly isolated fungal species.

## Introduction

Onychomycosis is most commonly caused by dermatophytes, such as *Trichophyton* species and *Epidermophyton floccosum*. Occasionally, it is associated with yeasts, such as *Candida* species; as well as other non-dermatophytic moulds, including mainly *Scopulariopsis brevicaulis*, *Hendersonula toruloidea*, *Aspergillus* species, *Acremonium* species and *Fusarium* species, which account for a few per cents to around 20% of all cases of onychomycosis in some series [1–5]. Due to the recent use of molecular technologies for identification of fungi, fungal species that have never been reported to be isolated from nails, including novel fungal species, are now recognized to be causes of onychomycosis. For example, in our study on the spectrum of *Exophiala* infections, we described the first reported cases of onychomycosis caused by *E. bergeri*, *E. oligosperma* as well as a novel *Exophiala* species, *E. hongkongensis* [6]. In another recent study, we also described another novel, onychomycosis-causing fungal species, *Aspergillus hongkongensis* [7].

As a result of our experience on the diversity of *Exophiala* and *Aspergillus* species associated with nail infections, we hypothesized that there is a previously unrecognized spectrum of fungi associated with onychomycosis. Furthermore, this unrecognized spectrum may also include potentially novel fungal species. To test these hypotheses, we performed genotypic identification on 24 phenotypically non-dermatophyte and non-*Aspergillus* fungal isolates recovered from finger and toe nails of patients with onychomycosis in four hospitals in Hong Kong. All these 24 fungal isolates were difficult-to-identify by conventional microscopic examination of lactophenol cotton blue stained adhesive tape preparations of the fungal colonies. *In vitro* susceptibilities of these 24 strains to 11 different antifungal agents were also characterized.

## Materials and methods

### Patients and fungal isolates

A total of 24 phenotypically non-dermatophyte and non-*Aspergillus* mould isolates, recovered from nail specimens collected during October 2006 to May 2016, were sent from four different hospitals in Hong Kong. All specimens were collected, transported, handled and processed according to the guidelines by the Clinical Laboratory Standards Institute (CLSI) [8]. Specifically, nails of the patients were cleaned with 70% alcohol prior to sample collection. Surface scrapings of the nails were then discarded, and deep area scrapings and debris were collected and transported in clean envelopes or sterile tubes. All subsequent work involving the processing and inoculation of the specimens was performed in a Class II biosafety cabinet in order to avoid possible environmental contamination. Moulds grown on the primary inoculation sites after direct inoculation of the nail samples on Sabouraud dextrose agar (SDA) (Oxoid, UK) supplemented with chloramphenicol (50 µg/ml) (Calbiochem, La Jolla, CA) were isolated, whereas any other mould present in other area of the culture plates were regarded as contaminants and discarded. These mould strains exhibited unrecognized morphologies and species level identification could not be confidently made by the clinical laboratories. All clinical data of the patients were collected by retrieving and analysing the patients’ hospital records. This study was approved by the Institutional Review Board of The University of Hong Kong/Hospital Authority. The reference strains *Aspergillus flavus* ATCC 204304, *Aspergillus fumigatus* ATCC 204305, *Candida parapsilosis* ATCC 22019^T^ and *Pichia kudriavzevii* (synonym: *Candida krusei*) ATCC 6258^T^ were obtained from the American Type Culture Collection (ATCC), USA.

### Molecular characterization

The partial 28S nuclear ribosomal DNA (nrDNA) and internal transcribed spacer (ITS) region were used as the primary markers for fungal identification. The partial translation elongation factor 1α gene (*tef1a*) was also sequenced since it was proposed as the secondary barcode for fungi [9]. In addition, partial actin gene (*act*) and partial β-tubulin gene (*benA*) sequencing was performed for *Cladosporium* and *Penicillium* strains, respectively. Extraction of fungal DNA, polymerase chain reaction (PCR) and sequencing of the partial 28S nrDNA, ITS, partial *tef1a*, partial *act* and/or partial *benA* for the case isolates were carried out following our previous publication [6] with the primer pairs ITS1/ITS4 [10], NL1/NL4 [11], EF1-1018F/EF1-1620R or Al33_alternative_f/EF1-1620R [9], ACT-512F/ACT-783R [12] and bt2a/bt2b [13], respectively. The DNA sequences obtained were then analysed by local alignment against sequences from the DDBJ/ENA/GenBank databases using BLAST for fungal identification. In addition, these DNA sequences, together with those of other closely related species accessioned in the DDBJ/ENA/GenBank databases, Q-bank [14] or Biological Resource Center, National Institute of Technology and Evaluation (NBRC), Japan, were then analysed by multiple sequence alignment using MUSCLE 3.8 [15]. After end-trimming, divergent or poorly aligned regions of the DNA sequences were removed using Gblocks 0.91b [16,17] with relaxed parameters. Tests for substitution models and phylogenetic tree reconstruction were performed by the maximum likelihood method using MEGA 6.0.6 [18].

### Antifungal susceptibility test

The *in vitro* susceptibilities against amphotericin B (Cayman Chemical, Ann Arbor, MI), anidulafungin (TargetMol, Boston, MA), caspofungin (TargetMol), micafungin (TargetMol), fluconazole (TargetMol), isavuconazole (TargetMol), itraconazole (Sigma-Aldrich), posaconazole (Sigma-Aldrich), voriconazole (TargetMol), flucytosine (TargetMol) and terbinafine (Sigma-Aldrich) (test range: 0.0156–8 mg/L for itraconazole and posaconazole; 0.0312–16 mg/L for other drugs) were determined by the microbroth dilution method according to the guidelines by the European Committee on Antimicrobial Susceptibility Testing [19]. Briefly, All the drugs were dissolved in sterile dimethyl sulphoxide (Sigma-Aldrich) for the preparation of stock solutions (3.2 g/L), which were stored in polypropylene vials (Axygen Scientific, Union City, CA) at −80°C until use. For the preparation of microdilution plates, the antifungal agent stock solutions were diluted using double-strength RPMI 1640 medium (Gibco, Grand Island, NY) buffered with 3-(N-morpholino)propanesulfonic acid (MOPS) (Gibco) supplemented with 2% glucose (BDH Chemicals, UK; w/v) and for each antifungal agent a dilution series at two times the final concentrations was produced and dispensed into the flat-bottomed wells of tissue culture-treated polystyrene 96-well microdilution plates (Wuxi NEST Biotechnology, China). The microdilution plates were sealed and stored at −80°C until use. For the preparation of inoculum, conidia were harvested from fungal cultures on SDA incubated at 25°C for 5–7 days and then resuspended in 0.1% Tween 20 (Sigma-Aldrich). The conidial suspensions were then filtered using cell strainers with a pore size of 10 µm (pluriSelect, Germany) to remove large hyphal fragments. The turbidity of each of the conidial suspension was then adjusted to 0.5 McFarland standard, and the conidial suspensions were then diluted ten times with sterile distilled water before being inoculated into the wells of the microdilution plates. The inoculated plates were incubated at 25°C, 30°C or 35°C, depending on the maximum growth temperature of the strains. Test results were read on days 2, 3 and/or 6 post-inoculation, depending on the growth rate of the strains. For echinocandins, minimum effective concentration (MEC) endpoints were recorded as the lowest drug concentrations in which abnormal, short and branched hyphal clusters were observed; whereas for the other antifungal agents, minimum inhibitory concentration (MIC) endpoints yielding no visible fungal growth by eyes were recorded. *Aspergillus flavus* ATCC 204304, *Aspergillus fumigatus* ATCC 204305, *Candida parapsilosis* ATCC 22019^T^ and *Pichia kudriavzevii* ATCC 6258^T^ were used as quality controls.

### Nucleotide sequence accession numbers

The partial 28S nrDNA, ITS, partial *tef1a*, partial *act* and/or partial *benA* sequences of the case isolates have been deposited to the DDBJ/ENA/GenBank databases. The nucleotide accession numbers are listed in Supplementary Table 1.

## Results

### Patient characteristics

The clinical characteristics of the 24 patients with phenotypically non-dermatophyte and non-*Aspergillus* mould isolated from nails are shown in Table 1. Except for one patient whose demographic information was not available, 15 (65.2%) of the remaining 23 patients were males while eight (34.8%) were females. The ages of the 23 patients ranged from 4 to 75 years, with a median age of 51 years. Out of the 22 patients with traceable clinical histories, 14 (63.6%) possessed predisposing underlying diseases, most frequently diabetes mellitus, hyperlipidaemia and hypertension, which may have made them more prone to the infections. Among the 18 patients with retrievable information on the nails involved, six (33.3%) and 12 (66.7%) patients had their fingernails and toenails involved, respectively. The nails affected in the other four patients were not specified.

**Table 1.** Cases of onychomycosis caused by non-dermatophytic, non-*Aspergillus* moulds reported in this study.

Strain	Sex^a^/age (year)	Underlying diseases	Nail involved	Molecular identification^c^			
				Species	Per cent identity by BLAST (closely matched strain)		
					28S nrDNA	ITS	*tef1a*
HKU40	F/47	Bilateral ovarian cyst & uterine fibroid	Left middle finger	Potentially novel *Trichomeriaceae* species	−	−	−
HKU41	M/48	Ventricular septal defect with repair	Toe	Potentially novel *Arthrinium* species	−	−	−
HKU42	M/55	None	Toe	Potentially novel *Paracremonium* species	−	−	−
HKU47	M/62	Hepatitis B, hepatocellular carcinoma, renal stones	Right index, middle & ring fingers	Potentially novel *Amorosiaceae* species	−	−	−
HKU56	M/49	N/A^b^	N/A^b^	Potentially novel *Sympoventuriaceae* species	−	−	−
HKU62	M/4	None	Left big toe	Potentially novel *Pyrenochaetopsis* species	−	−	−
HKU69	F/45	Paranoid schizophrenia/ temporal lobe epilepsy/ personality disorder	N/A^b^	Potentially novel *Penicillium* species	−	−	−
PW1843	M/44	Paraplegia with neurogenic bladder	Right big toe	*Acremonium egyptiacum*	100% (CBS 286.70B)	100% (CBS 286.70B)	100% (06239)
PW2467	F/52	Hypertension, endometriosis	Left ring finger	*Rhinocladiella similis*	100% (CBS 126848)	99.8% (CBS 111763^T^)	N/A^d^
PW2785	M/23	None	Left big toe	*Cephalotheca foveolata*	100% (NBRC 100905^T^)	99.0% (NBRC 100905^T^)	N/A^d^
PW2786	M/75	Hypertension	N/A	*Cephalotheca foveolata*	100% (NBRC 100905^T^)	99.0% (NBRC 100905^T^)	N/A^d^
PW2861	M/59	Hypertension	Right thumb	*Pseudopithomyces maydicus*	100% (CBS 491.88)	100% (MFLUCC 14-0391)	N/A^d^
PW2989	M/50	None	N/A^b^	*Didymella gardeniae*	100% (CBS 626.68^T^)	99.8% (CBS 626.68^T^)	N/A^d^
PW3024	N/A^b^	N/A^b^	N/A^b^	*Aspergillus keratitidis*	100% (BCRC 34221^T^)	100% (BCRC 34221^T^)	N/A^d^
PW3035	M/51	Hepatitis B	Finger	*Cladosporium halotolerans*	100% (CBS 127371)	99.8% (EXF-572^T^)	N/A^d^
PW3036	M/75	Diabetes mellitus, hypertension, hyperlipidaemia	Toe	*Cladosporium halotolerans*	100% (CBS 127371)	100% (EXF-572^T^)	N/A^d^
PW3038	M/48	None	Right big toe	*Chaetomium globosum*	100% (CBS 160.62^T^)	99.8% (CBS 160.62^T^)	99.8% (CBS 160.62^T^)
PW3041	F/46	None	Right ring finger	*Rhinocladiella similis*	100% (CBS 126848)	99.8% (CBS 111763^T^)	N/A^d^
PW3042	F/54	None	Right big toe	*Cladosporium lebrasiae*	N/A^d^	99.2% (UBOCC-A-112063^T^)	N/A^d^
PW3043	F/61	Hypertension, hyperlipidaemia, ischaemic heart disease	Big toe	*Microascus gracilis*	100% (CBS 369.70^T^)	100% (CBS 369.70^T^)	99.8% (CBS 369.70^T^)
PW3044	F/64	Diabetes mellitus, hypertension, carcinoma of sigmoid colon	Right big toe	*Exophiala oligosperma*	99.8% (CBS 725.88^T^)	99.6% (CBS 725.88^T^)	98.8% (CBS 725.88^T^)
PW3045	M/48	None	Big toe	*Purpureocillium lilacinum*	100% (ATCC 10114^T^)	100% (CBS 284.36^T^)	100% (CBS 284.36^T^)
PW3046	F/64	Hypertension, hyperlipidaemia	Right big toe	*Cladosporium halotolerans*	99.8% (CBS 127371)	99.6% (EXF-572^T^)	N/A^d^
PW3047	M/68	Diabetes mellitus, hypertension, hyperlipidaemia, ischaemic heart disease	N/A^b^	*Simplicillium obclavatum*	99.5% (CBS 311.74^T^)	100% (CBS 311.74^T^)	99.8% (CBS 311.74^T^)

^a^F, female; M, male.

^b^N/A, not available.

^c^28S nrDNA, 28S nuclear ribosomal DNA; ITS, internal transcribed spacer; *tef1a*, translation elongation factor 1 *α* gene.

^d^Sequence for the corresponding species not available in the DDBJ/ENA/GenBank databases.

### Molecular characterization

Of the 24 phenotypically non-dermatophyte and non-*Aspergillus* nail isolates, 16 were identified as 12 different known fungal species, namely *Acremonium eqyptiacum*, *Aspergillus keratitidis*, *Cephalotheca foveolata*, *Chaetomium globosum*, *Cladosporium halotolerans*, *Didymella gardeniae*, *Exophiala oligosperma*, *Microascus gracilis*, *Pseudopithomyces maydicus*, *Purpureocillium lilacinum*, *Rhinocladiella similis* and *Simplicillium obclavatum*, based on partial 28S nrDNA, ITS, and/or partial *tef1a* sequencing as well as phylogenetic analyses (Figure 1). These isolates exhibited ≥99.5% (28S nrDNA) and ≥99.0% (ITS) sequence identities with their respective species (Table 1). As for the partial *tef1a* sequences, only six out of these 16 isolates could be successfully identified as their respective species with sequence identities of ≥99.8% (Table 1). The *tef1a* sequences of the other ten isolates could not be matched to their respective species because there was a lack of respective sequence data in the DDBJ/ENA/Genebank databases.Figure 1.Phylogenetic trees showing the classification and relationship of the 24 nail isolates recovered in this study inferred from (a) partial 28S nuclear ribosomal DNA (nrDNA) (403 nucleotide positions of the trimmed sequence alignments), (b) internal transcribed spacer (ITS) region (468 nucleotide positions of the trimmed sequence alignments) and (c) partial *tef1a* (524 nucleotide positions of the trimmed sequence alignments) sequence data by the maximum likelihood method using the substitution models TN93 (Tamura-Nei model) + G (gamma-distributed rate variation) (28S nrDNA), K2 (Kimura 2-parameter model) + G + I (estimated proportion of invariable sites) (ITS) or TN93 + G + I (*tef1a*). The scale bars indicate the estimated numbers of substitutions per base. All names and accession numbers are given as cited in the DDBJ/ENA/GenBank databases. Numbers at nodes indicate levels of bootstrap support calculated from 1,000 trees and are expressed as percentage. Only nodes that were well supported (≥70% bootstrap support) have their bootstrap values shown. The 24 nail isolates were scattered across three different fungal classes (*Dothideomycetes*, *Eurotiomycetes*, and *Sordariomycetes*).

**Figure:**
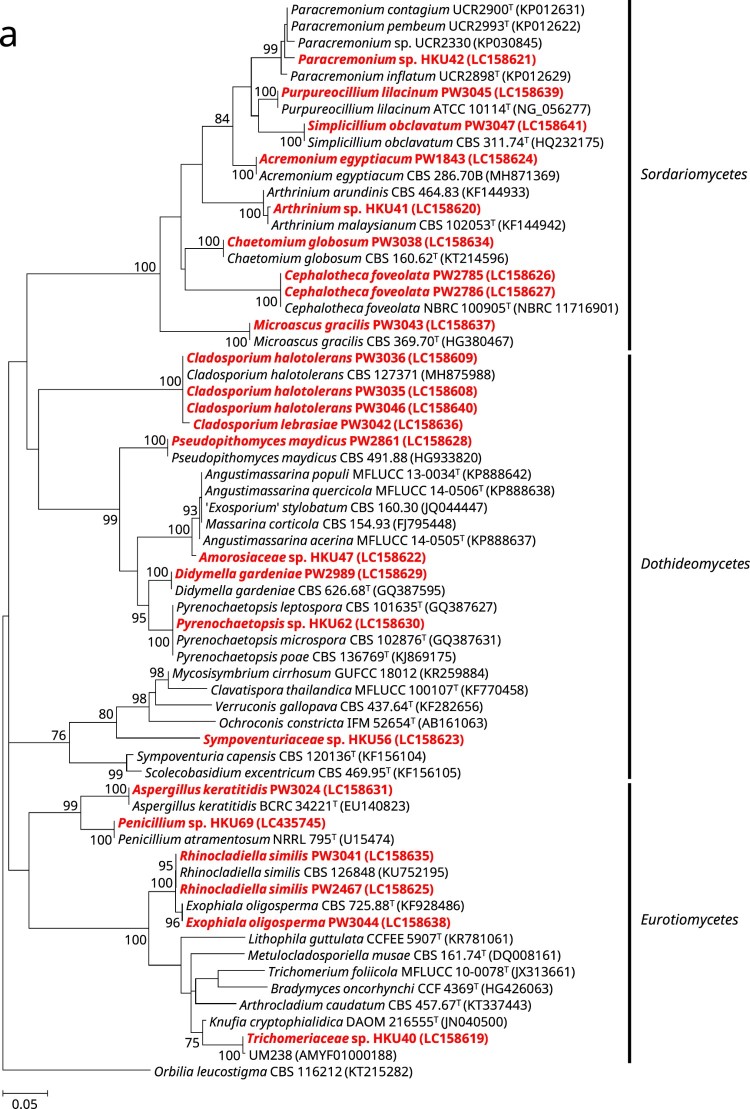

**Figure:**
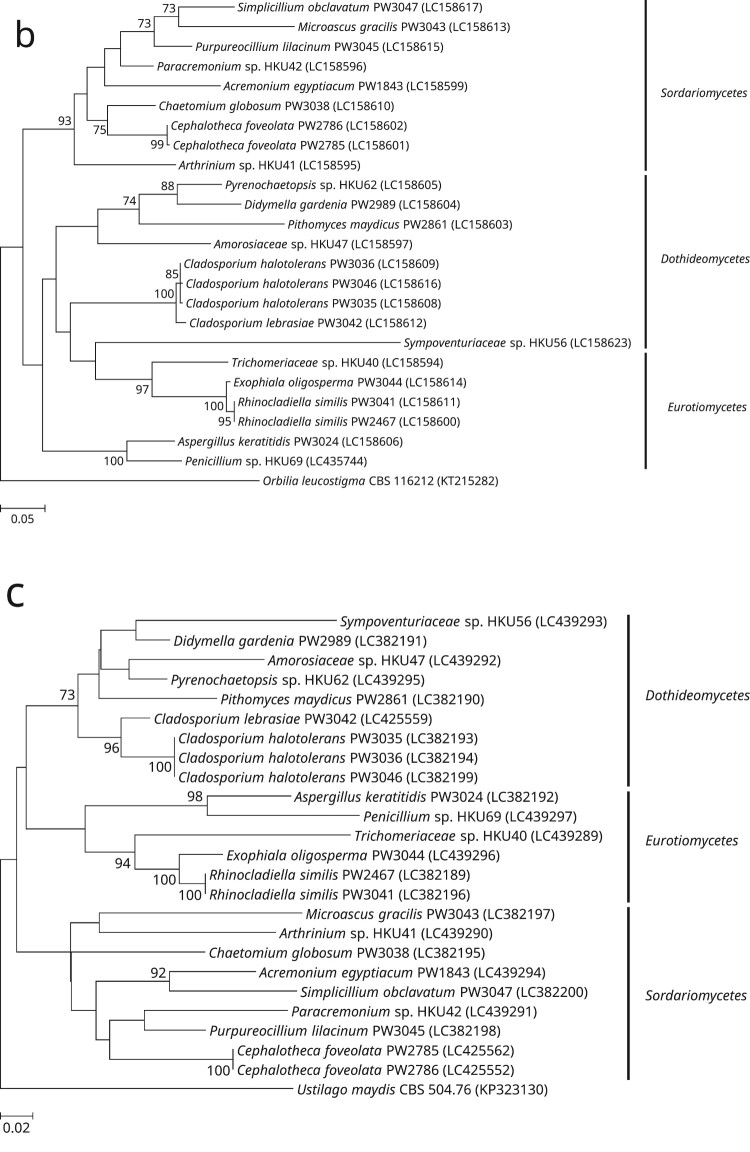

One (strain PW3042) of the 24 nail isolates could only be identified as a member of the *Cladosporium sphaerospermum* species complex using partial 28S nrDNA and ITS sequencing. BLAST analysis showed that this strain possessed ≥98.0% 28S nrDNA sequence identities to members of the *Cladosporium sphaerospermum* species complex, while the ITS sequence of this strain exhibited 99.6%, 99.2% and 98.6% identities to those of the ex-type strains of *Cladosporium dominicanum*, *Cladosporium lebrasiae* and *Cladosporium cycadicola*, respectively. In order to better resolve the species identity of strain PW3042, sequencing of an additional gene locus, partial *act*, was performed and the result showed that this strain possessed 97.9%, 94.8% and 89.2% identities to those of the ex-type strains of *Cladosporium lebrasiae*, *Cladosporium dominicanum* and *Cladosporium cycadicola*, respectively. Phylogenetic analysis using the concatenated ITS and partial *act* sequence showed that strain PW3042 was clustered with the ex-type strain of *Cladosporium lebrasiae* with high bootstrap support (Supplementary Figure 1), suggesting that it was a member of this species. Similar to the previous 16 nail isolates, the *tef1a* sequence of strain PW3042 could also not be matched to *Cladosporium lebrasiae* which was due to the lack of corresponding sequence data in the DDBJ/ENA/Genebank databases.

For the remaining seven nail isolates, their partial 28S nrDNA, ITS and *tef1a* sequences could not be used to reveal their definite species identities. BLAST analysis showed that strain HKU69 possessed 100% partial 28S nrDNA sequence identity to the ex-type strain of *Penicillium atramentosum* as well as 99.8%, 99.4% and 99.3% ITS sequence identities to the ex-type strains of *Penicillium mexicanum*, *Penicillium magnielliptisporum* and *Penicillium atramentosum*, respectively. This suggested that strain HKU69 is a member of *Penicillium* section *Paradoxa*; although *tef1a* sequencing showed that HKU69 possessed a 95.8% sequence identity to *Penicillium chrysogenum* strain MOS731, which belongs to section *Chrysogena* instead, and this might have been due to the fact that *tef1a* sequence data for section *Paradoxa* are not available in the DDBJ/ENA/GenBank databases. To further resolve the identity of strain HKU69, sequencing of an additional gene locus, partial *benA*, was performed and the result showed that there were 97.9%, 96.5% and 94.9% identities between the *benA* sequence of strain HKU69 and those of the ex-type strains of *Penicillium mexicanum*, *Penicillium magnielliptisporum* and *Penicillium atramentosum*, respectively. Phylogenetic analysis based on the *benA* sequences showed that strain HKU69 was clustered with, but distinct from *Penicillium mexicanum* (Supplementary Figure 2), suggesting that this strain may represent a novel *Penicillium* species in section *Paradoxa.* Similarly, phylogenetic characterization based on the ITS, partial 28S nrDNA and/or partial *tef1a* sequences showed that strains HKU41, HKU42 and HKU62 stood out as distinct branches in three different genera, namely *Arthrinium*, *Paracremonium* and *Pyrenochaetopsis*, respectively; and the three strains were the most closely related to, but distinct from, *Arthrinium malaysianum*, *Paracremonium contagium*/*Paracremonium pembeum* and *Pyrenochaetopsis microspora*, respectively (Supplementary Figures 3–5). This suggested that strains HKU41, HKU42 and HKU62 may represent novel species in these three genera. As for strains HKU40, HKU47 and HKU56, phylogenetic analyses showed that they were clustered with genera of the families *Trichomeriaceae*, *Amorosiaceae* and *Sympoventuriaceae*, respectively; and the three strains were the most closely related to, but distinct from, the genera *Knufia*, *Angustimassarina* and *Ochroconis*, respectively (Supplementary Figures 6–8). This suggested that strain HKU40, HKU47 and HKU56 may represent novel genera and species with these three families.

### In vitro antifungal susceptibilities

The MICs or MECs of the 24 nail isolates against 11 antifungal agents are listed in Table 2. Excluding strain HKU56 of which the results were only read on day 3 post-inoculation as well as strains HKU40 and PW3024 of which the results were read only on day 6 post-inoculation due to their slow growth, results for the remaining 21 nail isolates were examined on both days 2 and 3 post-inoculation. Results obtained from days 2 and 3 post-inoculation were generally in congruence (≤2 log_2_ difference) to each other, except for strain HKU69 against isavuconazole, strains PW2861 and PW3035 against itraconazole, strains HKU47, PW2785 and PW2786 against posaconazole, strain PW2785 against voriconazole, strains PW3044 against micafungin as well as strain PW2467 against flucytosine where there were ≥3 log_2_ difference between the MIC/MEC results obtained on days 2 and 3 post-inoculation. Despite these, it was observed that 66.7% of all the nail isolates tested possessed low MICs against posaconaozle (<1 µg/mL); whereas 95.8% and 87.5% of the nail isolates possessed high MICs against fluconazole (≥8 µg/mL) and flucytosine (≥4 µg/mL), respectively. It was also noted that two (strains HKU42 and PW3043) of the nail isolates possessed high MICs/MECs against all the antifungal agents tested.

**Table 2.** Minimum inhibitory concentrations or minimum effective concentrations of the phenotypically non-dermatophyte and non-*Aspergillus* moulds isolated in this study against 11 antifungal agents.

Incubation temperature	Strain	Minimum inhibitory concentrations (MICs) or minimum effective concentrations (MECs) (µg/mL)																																
		Triazoles															Echinocandins									Others								
		Fluconazole			Isavuconazole			Itraconazole			Posaconazole			Voriconazole			Anidulafungin			Caspofungin			Micafungin			Amphotericin B			Flucytosine			Terbinafine		
		D2	D3	D6	D2	D3	D6	D2	D3	D6	D2	D3	D6	D2	D3	D6	D2	D3	D6	D2	D3	D6	D2	D3	D6	D2	D3	D6	D2	D3	D6	D2	D3	D6
35°C	HKU42	>16	>16	–	16	16	–	>8	>8	–	>8	>8	–	4	4	–	>16	>16	–	>16	>16	–	>16	>16	–	2	2	–	>16	>16	–	8	16	–
	PW2785	>16	>16	–	8	16	–	>8	>8	–	1	8	–	0.5	8	–	0.25	0.25	–	0.03	0.03	–	0.125	0.125	–	2	4	–	>16	>16	–	1	4	–
	PW2786	>16	>16	–	4	>16	–	8	>8	–	1	>8	–	1	2	–	0.5	0.5	–	0.03	0.03	–	0.125	0.125	–	8	>16	–	>16	>16	–	1	>16	–
	PW2989	>16	>16	–	4	4	–	8	>8	–	0.5	0.5	–	2	4	–	>16	>16	–	16	16	–	>16	>16	–	1	2	–	>16	>16	–	1	4	–
	PW3043	>16	>16	–	16	>16	–	>8	>8	–	>8	>8	–	>16	>16	–	>16	>16	–	>16	>16	–	>16	>16	–	4	>16	–	>16	>16	–	8	16	–
	PW3047	>16	>16	–	0.5	1	–	>8	>8	–	0.25	0.5	–	0.25	0.25	–	>16	>16	–	16	>16	–	>16	>16	–	>16	>16	–	>16	>16	–	0.125	0.125	–
30°C	HKU40	–	–	0.06	–	–	0.03	–	–	0.015	–	–	0.015	–	–	0.03	–	–	0.03	–	–	0.25	–	–	0.03	–	–	0.03	–	–	>16	–	–	0.03
	HKU41	–	>16	–	–	2	–	–	>8	–	–	>8	–	–	16	–	–	0.03	–	–	0.5	–	–	0.03	–	–	0.25	–	–	>16	–	–	0.5	–
	HKU47	>16	>16	–	4	8	–	>8	>8	–	1	>8	–	0.5	1	–	0.03	0.03	–	1	2	–	8	16	–	0.25	1	–	4	8	–	0.06	0.125	–
	HKU56	–	8	–	–	0.125	–	–	0.03	–	–	0.015	–	–	0.06	–	–	0.03	–	–	0.25	–	–	0.03	–	–	0.5	–	–	1	–	–	0.03	–
	HKU69	>16	>16	–	0.25	>16	–	0.25	0.5	–	0.06	0.06	–	0.5	0.5	–	0.06	0.06	–	0.125	0.125	–	0.03	0.03	–	0.5	1	–	4	4	–	0.25	0.25	–
	PW1843	>16	>16	–	8	16	–	>8	>8	–	>8	>8	–	2	4	–	>16	>16	–	>16	>16	–	>16	>16	–	4	16	–	>16	>16	–	0.25	0.5	–
	PW2467	>16	>16	–	8	16	–	0.25	0.5	–	0.06	0.25	–	2	4	–	>16	>16	–	>16	>16	–	>16	>16	–	2	4	–	0.5	16	–	0.5	1	–
	PW2861	>16	>16	–	0.5	1	–	0.015	0.125	–	0.015	0.125	–	0.25	0.5	–	8	>16	–	2	4	–	4	8	–	0.5	1	–	4	16	–	0.125	0.5	–
	PW3024	–	–	>16	–	–	0.5	–	–	0.125	–	–	0.06	–	–	2	–	–	2	–	–	4	–	–	4	–	–	2	–	–	>16	–	–	0.25
	PW3035	>16	>16	–	4	8	–	0.25	2	–	0.125	0.25	–	1	2	–	2	2	–	4	4	–	8	8	–	2	>16	–	4	>16	–	1	2	–
	PW3036	>16	>16	–	4	4	–	0.25	0.5	–	0.06	0.125	–	2	2	–	8	8	–	8	8	–	8	16	–	16	>16	–	16	>16	–	0.5	1	–
	PW3038	>16	>16	–	1	1	–	0.25	0.5	–	0.25	0.25	–	1	1	–	0.25	0.25	–	8	16	–	0.25	0.25	–	1	1	–	>16	>16	–	16	>16	–
	PW3041	>16	>16	–	4	>16	–	0.25	0.5	–	0.06	0.125	–	1	4	–	>16	>16	–	>16	>16	–	2	2	–	2	4	–	0.25	1	–	0.25	1	–
	PW3042	>16	>16	–	4	4	–	0.06	0.06	–	0.015	0.015	–	1	1	–	0.25	0.25	–	4	4	–	0.03	0.03	–	0.5	0.5	–	4	16	–	1	1	–
	PW3044	16	>16	–	0.5	0.5	–	0.015	0.015	–	0.015	0.015	–	0.125	0.25	–	8	>16	–	>16	>16	–	0.5	16	–	1	2	–	0.25	1	–	0.06	0.125	–
	PW3045	>16	>16	–	2	2	–	>8	>8	–	0.5	1	–	1	1	–	>16	>16	–	>16	>16	–	>16	>16	–	>16	>16	–	>16	>16	–	1	2	–
	PW3046	>16	>16	–	4	4	–	0.25	0.25	–	0.125	0.125	–	1	2	–	0.25	0.25	–	2	4	–	0.25	0.5	–	2	2	–	16	>16	–	0.5	1	–
25°C	HKU62	>16	>16	–	2	4	–	0.06	.06	–	0.06	0.06	–	0.5	1	–	0.03	0.03	–	8	8	–	0.03	0.03	–	0.25	0.25		>16	>16	–	0.25	0.5	–

## Discussion

In this study, we showed that a high diversity of moulds was associated with onychomycosis. The most affected age group was 40–49 years (34.8%), followed by 50–59 years (26.1%) and 60–69 years (21.7%). This is similar to previous studies that nail infections due to non-dermatophytic moulds are the most prevalent in patients who were 40–69 years old [5,20]. It has been well reported that a large proportion of non-dermatophyte mould onychomycoses involve the big toes of the patients [5,21]. In the present study, 47.4% of the cases with information on the nails involved affected the big toes, in line with the literature. Among the 24 isolates in this study, 17 (71%) could be confidently identified to the species level using a combination of microscopic examination and DNA sequencing. These 17 isolates, representing 13 species, belonged to 11 different genera (*Acremonium*, *Cephalotheca*, *Chaetomium*, *Cladosporium*, *Didymella*, *Exophiala*, *Microascus*, *Rhinocladiella*, *Pseudopithomyces*, *Purpureocillium* and *Simplicillium*) and at least nine families (*Cephalothecaceae*, *Chaetomiaceae*, *Cladosporiaceae*, *Cordycipitaceae*, *Didymellaceae*, *Didymosphaeriaceae*, *Herpotrichiellaceae*, *Microascaceae* and *Ophiocordycipitaceae*; *Acremonium egyptiacum* is currently classified under *Hypocreales incertae sedis* with no familial assignment yet) (Table 1). It is of note that nine of these 13 species have been reported to cause nail infections [6,22–39] and most were also identified by DNA sequencing, although quite a number of the isolates were included in molecular epidemiology studies on specific fungal genera. However, these species in causing nail infections might have been under reported before the use of molecular identification methods. For example, four of the 17 cases in the present study were due to *Cladosporium* species (*Cladosporium halotolerans* and *Cladosporium lebrasiae*), which were usually reported as *Cladosporium sphaerospermum* species complex when the mould was identified morphologically. Similarly, one of the nail isolates recovered in this study were molecularly identified as *Exophiala oligosperma* and two as *Rhinocladiella similis*. These two species might have also been identified as *Exophiala jeanselmei*–*Exophiala spinifera* complex in previous reports. Interestingly, molecular identification revealed that one of the phenotypically non-dermatophyte and non-*Aspergillus* nail isolate recovered in this study (PW3024) was actually an aspergillus (*Aspergillus keratitidis* [synonym = *Sagenomella keratitidis*]). Phenotypically, *Aspergillus keratitidis* is a “*Phialosimplex*”-like fungus with simple monophialidic conidiogenous structures where conidia are produced in chains [40,41], which are very different from the vesiculate *Aspergillus* conidiophores. This fungus is also genetically related to *Phialosimplex*/*Polypaecilum* [40]. Recent phylogenetic analyses demonstrated that *Phialosimplex*/*Polypaecilum* were included in the monophyletic *Aspergillus sensus stricto* clade [42–44] and so were transferred as *Aspergillus* subgenus *Polypaecilum* [44]. Following this change “*Sagenomella*” *keratitidis*, despite lacking typical *Aspergillus* micromorphologies, was also renamed as *Aspergillus keratitidis* [45].

A significant proportion of the isolates were potentially novel fungal species. During our previous studies on the epidemiology of *Exophiala* and *Aspergillus* species, through sequencing multiple gene loci, in addition to the high species diversity observed, we also discovered two novel pathogenic fungi, *Exophiala hongkongensis* and *Aspergillus hongkongensis*, both recovered from patients with onychomycosis. As for the present study, among the 24 isolates recovered from nail infections, seven (29%) were potentially novel species. DNA sequencing showed that these isolates formed distinct branches on phylogenetic trees. Interestingly, these seven potentially novel species belong to four different known and three potentially novel genera of seven different families, in line with the high diversity of fungal species observed. Remarkably, a number of species closely related to these potentially novel genera and/or species are human and/or animal pathogens. For example, strains HKU41, HKU42 and HKU69 are potentially novel *Arthrinium*, *Paracremonium* and *Penicillium* species, respectively; and members of all these three genera have been reported as agents of mycoses [46–49] In particular, *Arthrinium arundinis* [50] and *Penicillium* species [2,51–53] have been associated with onychomycosis. Moreover, strains HKU40 and HKU56 are potentially novel genera and species in the families *Trichomeriaceae* and *Sympoventuriaceae*, respectively. Members of both these families are black yeast-like fungi and are well recognized agents of nail infections [54]. It is also of note that strain HKU40 (*Trichomeriaceae* sp.) is closely related to a Malaysian strain UM238, which was isolated from skin scraping [33]. As for strains HKU47 and HKU62, although *Amorosiaceae* and *Pyrenochaetopsis* species have not been reported to be associated with human infection, they are *Phoma*-like dematiaceous moulds under the order *Pleosporales*. Members of *Pleosporales*, especially *Phoma* species, are also known to cause nail/skin infections [33,55–57]. All these highlighted the potential of these novel strains in causing superficial infections, including onychomycosis.

To guide proper treatment, molecular identification and antifungal susceptibility testing should be performed for these uncommonly isolated fungal species. The first line treatment of finger or toe nail onychomycosis is oral terbinafine, itraconazole or fluconazole for months. In general, dermatophytes are susceptible to these antifungal agents. However, in the present study, it was observed that 12.5% (day 2 results [day 6 for strains HKU40 and PW3024]) or 33.3% (day 3 results [day 6 for strains HKU40 and PW3024]), 41.7% and 95.8% of the fungal isolates possessed MICs of >1 µg/mL to terbinafine, itraconazole and fluconazole, respectively. In addition, it is of note that two (HKU42 and PW1843) of the nail isolates characterized in this study possessed high MICs/MECs for most of the drugs tested. While the susceptibility results obtained in the current study for strain PW1843 was in line with those for *Acremonium egyptiacum* reported in a previous study where this species only possessed low MICs against terbinafine [30]; susceptibility data for *Paracremonium* species is not available in the literature. The lack of antifungal susceptibility testing results is also the case for most of the other mould species identified in the present study. Since these fungal species are uncommonly recovered from clinical samples and data of antifungal susceptibility testing results on these species are highly limited in the literature, it would be difficult to predict the susceptibility profile of individual species even when a particular strain is identified to the species level. Unfortunately, the details of the antifungal regimen and clinical response of the patients in this study could not be retrieved for further analysis.

## Supplementary Material

Supplemental Material

## Declaration

Part of this work has contributed to the PhD thesis by Chi-Ching Tsang submitted to The University of Hong Kong, Hong Kong.
