# Supplementary material for: Diversity of phenotypically non-dermatophyte, non-Aspergillus filamentous fungi causing nail infections: importance of accurate identification and antifungal susceptibility testing
Source: Emerg Microbes Infect. 2019 Apr 2;8(1):531–41. doi: 10.1080/22221751.2019.1598781 (PMC6455232; doi:10.1080/22221751.2019.1598781)
Supplement: Supplemental Material [file TEMI_A_1598781_SM9123.zip › Supplementary_Figures_revised_Legend_.docx]

**Fig. S1.** Phylogenetic tree showing the relationship of the nail isolates PW3035, PW3036, PW3042 and PW3046 recovered in this study with members of the *Cladosporium sphaerospermum* species complex inferred from the concatenated ITS and partial *act1* sequence data (626 nucleotide positions of the trimmed sequence alignments) by the maximum likelihood method using the substitution model K2 (Kimura 2-parameter model) + G (gamma-distributed rate variation) + I (estimated proportion of invariable sites). The scale bar indicates the estimated number of substitutions per base. All names and accession numbers are given as cited in the DDBJ/ENA/GenBank databases. Numbers at nodes indicate levels of bootstrap support calculated from 1,000 trees and are expressed as percentage. Only nodes that were well supported (≥70% bootstrap support) have their bootstrap values shown.

**Fig. S2.** Phylogenetic trees showing the relationship of the nail isolate HKU69 recovered in this study with members of *Penicillium* section *Paradoxa* inferred from the (a) ITS (532 nucleotide positions of the trimmed sequence alignments) and (b) partial *benA* (381 nucleotide positions of the trimmed sequence alignments) sequence data by the maximum likelihood method using the substitution models T92 (Tamura 3-parameter model) + G (ITS) or K2 + G (*benA*). The scale bar indicates the estimated number of substitutions per base. All names and accession numbers are given as cited in the DDBJ/ENA/GenBank databases. Numbers at nodes indicate levels of bootstrap support calculated from 1,000 trees and are expressed as percentage. Only nodes that were well supported (≥70% bootstrap support) have their bootstrap values shown.

**Fig. S3.** Phylogenetic trees showing the relationship of the nail isolate HKU41 recovered in this study with *Arthrinium* species inferred from the (a) partial 28S nrDNA (296 nucleotide positions of the trimmed sequence alignments) and (b) ITS (447 nucleotide positions of the trimmed sequence alignments) sequence data by the maximum likelihood method using the substitution models K2 + I (28S nrDNA) or K2 + G (ITS). The scale bar indicates the estimated number of substitutions per base. All names and accession numbers are given as cited in the DDBJ/ENA/GenBank databases. Numbers at nodes indicate levels of bootstrap support calculated from 1,000 trees and are expressed as percentage. Only nodes that were well supported (≥70% bootstrap support) have their bootstrap values shown.

**Fig. S4.** Phylogenetic trees showing the relationship of the nail isolate HKU42 recovered in this study with *Paracremonium* species inferred from the (a) partial 28S nrDNA (487 nucleotide positions of the trimmed sequence alignments), (b) ITS (421 nucleotide positions of the trimmed sequence alignments) and (c) partial *tef1a* (324 nucleotide positions of the trimmed sequence alignments) sequence data by the maximum likelihood method using the substitution model K2 + G. The scale bar indicates the estimated number of substitutions per base. All names and accession numbers are given as cited in the DDBJ/ENA/GenBank databases. Numbers at nodes indicate levels of bootstrap support calculated from 1,000 trees and are expressed as percentage. Only nodes that were well supported (≥70% bootstrap support) have their bootstrap values shown.

**Fig. S5.** Phylogenetic trees showing the relationship of the nail isolate HKU62 recovered in this study with *Pyrenochaetopsis* species inferred from the (a) partial 28S nrDNA (551 nucleotide positions of the trimmed sequence alignments) and (b) ITS (350 nucleotide positions of the trimmed sequence alignments) sequence data by the maximum likelihood method using the substitution model K2 + G. The scale bar indicates the estimated number of substitutions per base. All names and accession numbers are given as cited in the DDBJ/ENA/GenBank databases. Numbers at nodes indicate levels of bootstrap support calculated from 1,000 trees and are expressed as percentage. Only nodes that were well supported (≥70% bootstrap support) have their bootstrap values shown.

**Fig. S6.** Phylogenetic trees showing the relationship of the nail isolate HKU40 recovered in this study with *Trichomeriaceae* species inferred from the (a) partial 28S nrDNA (481 nucleotide positions of the trimmed sequence alignments) and (b) ITS (387 nucleotide positions of the trimmed sequence alignments) sequence data by the maximum likelihood method using the substitution model K2 + G. The scale bar indicates the estimated number of substitutions per base. All names and accession numbers are given as cited in the DDBJ/ENA/GenBank databases. Numbers at nodes indicate levels of bootstrap support calculated from 1,000 trees and are expressed as percentage. Only nodes that were well supported (≥70% bootstrap support) have their bootstrap values shown.

**Fig. S7.** Phylogenetic trees showing the relationship of the nail isolate HKU47 recovered in this study with *Amorosiaceae* species inferred from the (a) partial 28S nrDNA (458 nucleotide positions of the trimmed sequence alignments) and (b) ITS (393 nucleotide positions of the trimmed sequence alignments) sequence data by the maximum likelihood method using the substitution model K2 + G. The scale bar indicates the estimated number of substitutions per base. All names and accession numbers are given as cited in the DDBJ/ENA/GenBank databases. Numbers at nodes indicate levels of bootstrap support calculated from 1,000 trees and are expressed as percentage. Only nodes that were well supported (≥70% bootstrap support) have their bootstrap values shown.

**Fig. S8.** Phylogenetic trees showing the relationship of the nail isolate HKU56 recovered in this study with *Sympoventuriaceae* species inferred from the (a) partial 28S nrDNA (482 nucleotide positions of the trimmed sequence alignments) and (b) ITS (354 nucleotide positions of the trimmed sequence alignments) sequence data by the maximum likelihood method using the substitution models TN93 + G + I (28S nrDNA) or T92 (Tamura-Nei model) + G (ITS). The scale bar indicates the estimated number of substitutions per base. All names and accession numbers are given as cited in the DDBJ/ENA/GenBank databases. Numbers at nodes indicate levels of bootstrap support calculated from 1,000 trees and are expressed as percentage. Only nodes that were well supported (≥70% bootstrap support) have their bootstrap values shown.
