# Supplementary material for: Diversity of phenotypically non-dermatophyte, non-Aspergillus filamentous fungi causing nail infections: importance of accurate identification and antifungal susceptibility testing
Source: Emerg Microbes Infect. 2019 Apr 2;8(1):531–41. doi: 10.1080/22221751.2019.1598781 (PMC6455232; doi:10.1080/22221751.2019.1598781)
Supplement: Supplemental Material [file TEMI_A_1598781_SM9123.zip › Table_S1.docx]

**Supplementary Table S1** DDBJ/ENA/GenBank accession numbers for the nucleotide sequences of the case isolates characterized in this study

|  | Locus | | | | |
| --- | --- | --- | --- | --- | --- |
| Strain | 28S nrDNA | ITS | *tef1a* | *act* | *benA* |
| HKU40 | LC158619 | LC158594 | LC439289 | − | − |
| HKU41 | LC158620 | LC158595 | LC439290 | − | − |
| HKU42 | LC158621 | LC158596 | LC439291 | − | − |
| HKU47 | LC158622 | LC158597 | LC439292 | − | − |
| HKU56 | LC158623 | LC158598 | LC439293 | − | − |
| HKU62 | LC158630 | LC158605 | LC439295 | − | − |
| HKU69 | LC435745 | LC435744 | LC439297 | − | LC440465 |
| PW1843 | LC158624 | LC158599 | LC439294 | − | − |
| PW2467 | LC158625 | LC158600 | LC382189 | − | − |
| PW2785 | LC158626 | LC158601 | LC425562 | − | − |
| PW2786 | LC158627 | LC158602 | LC425552 | − | − |
| PW2861 | LC158628 | LC158603 | LC382190 | − | − |
| PW2989 | LC158629 | LC158604 | LC382191 | − | − |
| PW3024 | LC158631 | LC158606 | LC382192 | − | − |
| PW3035 | LC158632 | LC158608 | LC382193 | LC164944 | − |
| PW3036 | LC158633 | LC158609 | LC382194 | LC164945 | − |
| PW3038 | LC158634 | LC158610 | LC382195 | − | − |
| PW3041 | LC158635 | LC158611 | LC382196 | − | − |
| PW3042 | LC158636 | LC158612 | LC425559 | LC164946 | − |
| PW3043 | LC158637 | LC158613 | LC382197 | − | − |
| PW3044 | LC158638 | LC158614 | LC439296 | − | − |
| PW3045 | LC158639 | LC158615 | LC382198 | − | − |
| PW3046 | LC158640 | LC158616 | LC382199 | LC164947 | − |
| PW3047 | LC158641 | LC158617 | LC382200 | − | − |
